# Research Progress in Abrasive Water Jet Processing Technology

**DOI:** 10.3390/mi14081526

**Published:** 2023-07-29

**Authors:** Hongqi Wang, Ruifu Yuan, Xinmin Zhang, Penghui Zai, Junhao Deng

**Affiliations:** 1 School of Mechanical and Power Engineering, Henan Polytechnic University, Jiaozuo 454003, China; 2 School of Energy Science and Engineering, Henan Polytechnic University, Jiaozuo 454003, China; 3 State Collaborative Innovation Center of Coal Work Safety and Clean-Efficiency Utilization, Jiaozuo 454003, China

**Keywords:** abrasive water jet, special processing, mechanism of erosion, process parameters, machining removal

## Abstract

Abrasive water jet machining technology is an unconventional special process technology; its jet stream has high energy, and its machining process is characterized by no thermal deformation, no pollution, high applicability, and high flexibility. It has been widely used for processing different types of materials in different fields. This review elaborates on the basic principles and characteristics of abrasive water jet processing, the mechanism of erosion, the simulation of the processing, the influence of process parameters in machining removal, and the optimization of improvements, as well as introduces the current application status, new technology, and future development direction of abrasive water jet technology. This review can provide an important information reference for researchers studying the machining processing of abrasive water jet technology.

## 1. Introduction

Abrasive water jet technology is derived from water jet technology and is based on the addition of abrasives to water jets to form an abrasive water jet. In 1979, the first abrasive water jet machining processes was invented by Hashish, which significantly increased the cutting capacity and range of applications compared with pure water jetting and laid the foundation for the development of abrasive water jetting [1]. Subsequently, the development of abrasive water jets has also become increasingly important to many countries, such as the United States, Europe, and Japan; in the last 30 years, abrasive water jet technology moved into a rapid development stage, while domestic research and the introduction of abrasive water jet machining technology and equipment also begun, such as in Chongqing University, China University of Mining and Technology, China University of Petroleum, and other research institutions that have conducted relevant research on this technology and achieved fruitful results. At present, the international developed ultrahigh-pressure equipment has reached pressures of 600 to 700 Mpa, further enhancing the processing capacity and efficiency, while with the increased accuracy of computer-controlled power transmission devices, such as the emergence of five-axis and six-axis machining systems, it has the ability and precision to machine complex surfaces in space. Abrasive water jet technology is a high-speed jet containing abrasive particles that is ejected through small holes in the nozzle and applied to the surface of the workpiece being machined, removing the material to be machined mainly by the high-speed collision and shear-slip effect of the abrasive particles [2]. Abrasive water jet technology represents a relatively new of unconventional special machining technology, which is a cold-working technology with a unique lack of heat deformation and heat impact, allowing many of the material’s own defects to be ignored during processing. Abrasive water jet technology is used in the processing of all types of materials and complex curved shapes due to its high processing capacity and flexibility. It has been well used in fields such as mechanical processing, surface treatment, construction engineering, precision instrumentation components, and aerospace and medical devices, and it plays a unique role in the treatment of flammable and explosive hazardous materials, chemical fiber, rubber, thermosensitive ceramics, and other heat-sensitive materials [3,4]. With the increasing development of science and technology to put forward higher requirements for processing efficiency and processing quality, more researchers are attracted by the advantages of abrasive water jet technology, such as adaptability, low processing forces, and nonpolluting, to conduct in-depth research on its processing process [5,6,7,8].

## 2. Basic Principles and Characteristics of Abrasive Water Jet Technology Processing

### 2.1. Basic Principles of Abrasive Water Jet Technology Processing

The abrasive water jet (AWJ) is a development from pure water jets, and it uses high-pressure water as a transport medium carrying tiny abrasive particles, which are clustered and spurted from tiny nozzles to form a high-energy solid–liquid mixed jet beam. The processing process of abrasive water jetting is to form high-energy jet beams to act on the workpiece to be machined and the mutual conversion of energy through impact to achieve the removal of the workpiece to be machined.

**Figure 1:**
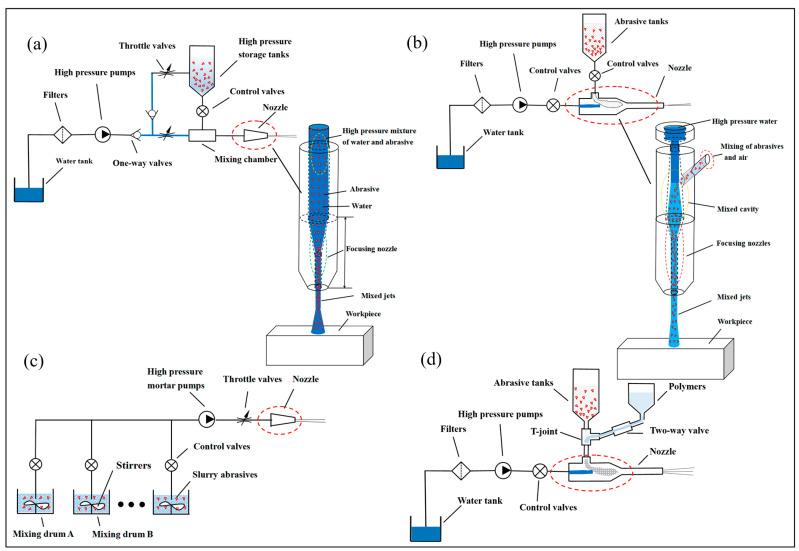
Principle diagram of abrasive water jet: (**a**) Premixed abrasive water jets. (**b**) Postmixed abrasive water jets. (**c**) Slurry abrasive water jet. (**d**) Abrasive water slurry jet.

Currently, there are many classification methods for abrasive water jets, which can be based on pressure levels, jet processing methods, jet medium, working environment, and the mixing method of the abrasive. Now, combined with the actual application of engineering, the following types are introduced with the classification of abrasive mixing methods. Abrasive water jets are divided into premixed abrasive water jets and postmixed abrasive water jets, depending on the method of abrasive is mixed. Premixed abrasive water jets are where the abrasive is fully mixed with the high-pressure water before it is accelerated, and then the abrasive is accelerated together by the high-pressure water and spurted out [9], as shown in Figure 1a. Its working principle is that the high-pressure pump pressurizes the filtered water and then enters the high-pressure storage tank through the one-way valve to mix the abrasive with the high-pressure water in advance, forming a high-pressure abrasive water jet, which is eventually sprayed out through the nozzle. The bypass waterway is to regulate and control the volume fraction of abrasive and water in the abrasive water jet. A postmixing abrasive water jet is a high-pressure water jet that enters the nozzle inlet through a pipe, mixes with the abrasive in the nozzle mixing chamber, and then accelerates to form a high-pressure abrasive water jet, finally spraying out through the nozzle [10], as shown in Figure 1b. If an appropriate proportion of additives are added to the water and abrasive mixture, an abrasive water slurry jet (AWSJ) can be made, which is then sprayed through the nozzle to form a water jet with a strong abrasive slurry set [3] according to the mixing methods, which are divided into premixed abrasive water slurry jets and postmixed abrasive water slurry jets, as shown in Figure 1d. In recent years, in order to make the abrasive and water mixing more uniformity, drawing on high-pressure slurry/grouting technology, the slurry abrasive water jet (S-AWJ) is proposed on the basis of the premixed abrasive water jet [11]; this means that the abrasive is premixed with water to form a slurry, and then the slurry is pressurized directly by a high-pressure mortar pump/slurry pump and shot from the nozzle, as shown in Figure 1c.

### 2.2. The Characteristics of Abrasive Water Jet Machining

Abrasive water jets have different processing characteristics depending on the mixing method. In the formation of the premixed abrasive water jet, the water is mixed with the abrasive in advance, and because the system is sealed, there is no air infiltration during the formation of the jet, which forms a solid–liquid two-phase high-pressure abrasive water jet [12]. In the pre-mixing, water and abrasive distribution between the more uniform, in the process of acceleration due to turbulence caused by the abrasive material between the mutual collision is small, its abrasive acceleration process is more adequate; the speed of the accelerated abrasive can be almost close to the speed of water, and the formation of jet beams with a certain bunching property is not easy to dispersal, the same conditions under the same parameters of the cutting ability is relatively strong. Because the diameter of the jet beam is small, it is commonly used for cutting objects, perforation, and other applications, for example, cutting steel plates, punching workpieces, etc. However, the mixed jet has high energy, causing some wear and tear on the equipment (e.g., booster equipment and nozzles); because the abrasive in the pre-mixing process is stored in high-pressure tanks and withstands the same high pressure as high-pressure water, the volume is limited, and in the process of using the abrasive tank, it needs to be opened periodically to add abrasive, so the continuous working time is relatively limited and the application of premixed abrasive water jets in practice is somewhat limited. During the formation of the postmixed abrasive water jet, water is pressurized by a high-pressure pump and enters the nozzle to form a high-speed water jet beam, causing a vacuum to form in the mixing chamber and sucking in the abrasive particles in the abrasive tank, and while mixing with each other under the impact of the high-pressure water flow, the jet is accelerated in the nozzle and then injected [13]. Due to the presence of air in the abrasive tank, the water jet in the mixing chamber also infiltrates air during the mixing of the abrasive, thus forming a three-phase fluid of water, abrasive, and air. It is simple in construction, has almost no influence on the pressurizing equipment, and it is easy to obtain high erosion energy by increasing the pressure. Therefore, it is widely used in industry considering the cost of the equipment. However, compared with the premixed method, its mixing process is not uniform, the air it draws in makes the turbulent dissipative effect of the jet increased, energy consumption increases, and compared with the same parameters of the premixed abrasive water jet erosion, the ability is relatively weak but also makes the air mixed with the jet of the cluster weak and easy to form dispersion, which makes it easier to cause quality defects during processing, such as kerf defects and cut sections. The abrasive slurry water jet is based on the abrasive water jet mixed with additives to form a four-phase fluid jet of water, abrasive, air, and polymer [5,14,15,16]. The premixing of the abrasive with the additive leaves the abrasive in suspension in the mixture, which reduces the frictional resistance between the jet and the pipe wall during the acceleration process after mixing with the high-pressure water, improving the stability, clustering, and coherence of the jet and enhancing the erosion capacity of the jet. Compared with the postmixing abrasive water jet with the same process parameters, abrasive slurry water jets have a higher power density, a higher processing capacity, and a better processing effect during processing, commonly used in applications such as polishing, cutting, rock breaking, and surface treatment of objects. The addition of additives gives the jet non-Newtonian fluid properties and allows the performance of the jet to be changed by varying the concentration of the additives; therefore, different processing effects can be obtained by adding different types of additives under the same working conditions. As the additives (water capacitive polymer) are mostly polymers of high molecular weight, they can increase the viscosity to reduce the turbulent effects of the jet. But the abrasive slurry water jet also has certain disadvantages, especially rapid stopping and starting and easily caused slurry liquid precipitation inside the pipeline and nozzle, resulting in long switching time, as well as inability to quickly stop and start, abrasive wear, etc. It is used relatively often in micromachining and polishing technology, where the working pressure of the jet is not high; its wide industrial application is somewhat restricted. The slurry abrasive water jet is based on the former mixed abrasive water jet, where a certain proportion of water and abrasive are stored in a certain number of slurry storage tanks under atmospheric pressure in advance, and the abrasive is mixed with water to form a slurry abrasive by means of an agitator, which is constantly stirred to prevent the abrasive from settling. The slurry is directly pressurized by a high-pressure mortar pump/mud pump and then ejected from the nozzle to form a solid–liquid two-phase high-pressure abrasive water jet. Compared with premixed abrasive water jets, the mixing of water and abrasive is carried out at atmospheric pressure, without the need for high-pressure sand storage tanks and by using multiple mixing drums together to solve the problems of the former mixed abrasive water jets, which are not able to continuously add sand, as well as the short duration of work and the risks and complex operating procedures of using high-pressure sand storage tanks, while the connection of a high-pressure hose between the nozzle and the high-pressure slurry pump greatly increases the flexibility and the range of use, making it suitable for processing long distances or large objects,, for example, in fire rescue, mineral extraction, workpiece cutting, etc. However, as the slurry abrasive water jet (S-AWJ) has high wear and tear on the slurry abrasive, as it flows through components such as high-pressure pumps, high-pressure pipelines, and nozzles, methods of wear reduction need to be explored, thus limiting the widespread industrial application of the technology.

## 3. Abrasive Water Jet Processing Material Mechanism

Abrasive water jet processing is the use of high-speed jet beam impact on processed material to achieve the material removal process, and therefore, the removal mechanism is based on the principle of erosion, and the impact of abrasive particles in the process of material removal by abrasive water jet plays the dominant role [17,18] in almost all of the removal tasks, for example, the removal of high hardness and high strength in difficult to machine materials; however, in the removal of low hardness and low strength in easily machinable materials, high-speed water jets can also achieve a certain amount of damage removal tasks. When the abrasive material impacts the surface of the object to be machined at a very high speed under the acceleration of the water flow, a very strong impact is produced, the removal of material is achieved in the form of cutting, fatigue, and fracture of the machined surface by abrasive particles. Cutting deformation wear occurs mainly when the sharp angular surface of the abrasive grain impacts the machined surface, while ploughing deformation wear occurs when the rounded surface of the abrasive grain impacts the machined surface. As a result, the machining mechanism differs for each material. Generally, researchers classify materials for machining as having brittle, plastic, or compound properties.

### 3.1. Processing Removal Mechanism of Brittle Materials

**Figure 2:**
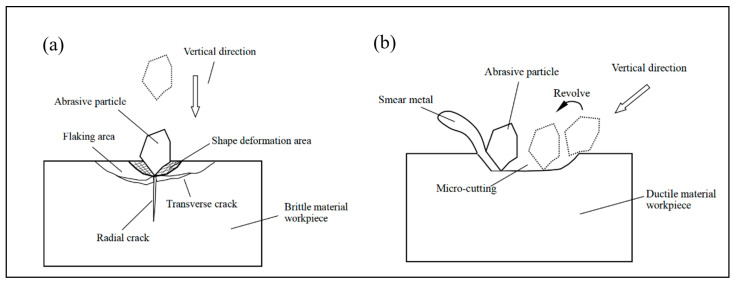
(**a**) damage removal of brittle materials by abrasive particles; (**b**) damage removal of ductile materials by abrasive particles.

**Figure 3:**
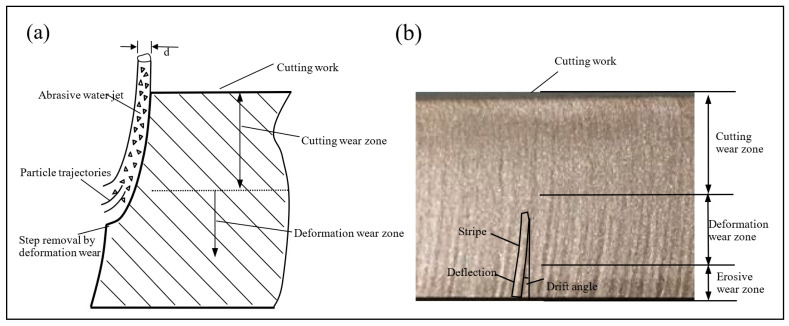
(**a**) formation process of steps in cutting; (**b**) sectional zoning of cutting actual topography.

It is generally accepted that the removal process of brittle materials is mainly achieved through the impact of abrasive particles on the processed material to produce microcracks. The cracking formation process of microcracks mainly includes radial cracks, transverse cracks, tapered cracks, intergranular and through crystal cracks, ring fracture cracks, mechanical micromachining, and mixed-mode damage. During the processing of brittle materials by abrasive water jets, the impact of abrasive particles on the machined surface satisfies the theory of indentation fracture [19]. When the abrasive particles impact the surface, a plastic deformation zone will be created in the area of contact between the particles and the surface under the action of compressive stress, and as the compressive stress increases, the plastic deformation zone will continue to increase, and when the tensile strength of the processed material is exceeded, a radial crack will be created perpendicular to the processed surface and expand downwards gradually away from the bottom of the plastic deformation zone. During the expansion of radial cracks, transverse cracks (lateral cracks) appear at the base of the plastic deformation of the machined material and expand parallel to the machined surface, eventually producing brittle spalling and achieving the destruction of the removed material. Radial cracking affects the integrity of the machined surface, and lateral cracking determines the volume of material to be removed, as shown in Figure 2a [20]. Brittle erosion is usually applied to brittle materials such as rocks, ceramics, glass, hard metals, etc., which break and shatter due to jet impact; in the process of brittle material erosion removal [21], the removal of the processed material is achieved by crack expansion and cutting occurring through contact stresses generated by abrasive particles impacting on the processed surface, where the jet impact angle has an influence on the removal mechanism of the processed material. The jet inclination angle is related to the highly efficient ductile shearing action in the machined material, which limits fracture marks and incomplete chip removal from the machined surface [22,23]. From the macroscopic mechanism of material removal, Hashish [24] proposed a step theory of cutting surface formation, as shown in Figure 3a, which divides the cutting surface into initial cutting zone, smooth cutting zone, and rough cutting zone. The cutting mechanism can be classified into cutting wear, deformation wear, and erosion wear by the quality of the surface cut, as shown in Figure 3b. From the top to the bottom of the cut surface of the material being machined, the degree of plastic deformation gradually increases.

**Figure 4:**
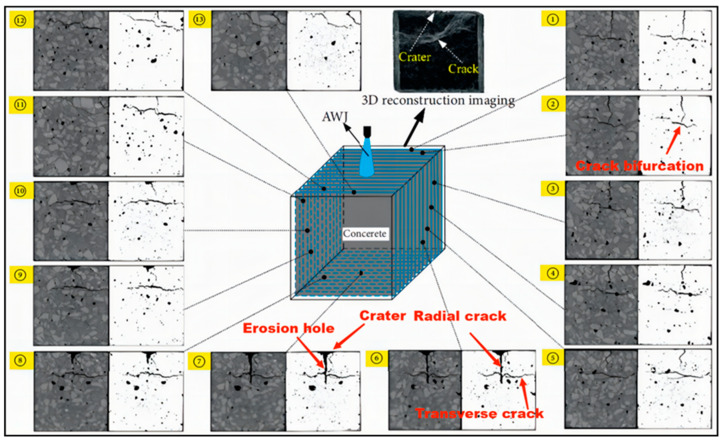
The propagation path of cracks in the concrete after abrasive water jet impact in the interior from the distribution and scanning results of CT sections [27]; Serial numbers ①–⑬ represent 13 typical CT slices obtained in the radial direction of the jet.

In observing the removal process of brittle materials, researchers have used a series of studies to determine the mechanism of separation of processed materials, including brittle fracture and plastic deformation phenomena. There were clearly visible scratches at smaller jet impact angles, as well as some degree of fracture between crystals [25,26]. It is shown that the angle of impact of the jet has an important influence on the fracture removal achieved between the crystals of the processed material. Traces of plastic deformation are also present but to a relatively small extent. In the cutting and grinding process of brittle materials such as ceramics, rocks, and concrete, it was found that the material breakage and spalling was a result of the combined effect of fatigue damage caused by the impact of the jet and water wedge. Liu et al. [27] found the crack expansion law crushing and removal mechanism during the crushing and removal process of abrasive water jet impact on concrete; in the shear region, there is a large number of cross cracks for the crack-dense area, where the region is mainly shear damage, compared with a small number of cracks in the tensile region, where the region is mainly tensile fracture damage, as shown in Figure 4. Chen et al. [28] also found the same when analyzing the damage area of jet-impacted concrete. The researchers found that when impacting concrete with different jet angles, when impacting at small inclination angles, the erosion process was dominated by microcutting, as well as when impacting at large inclined angles by spalling or medium inclined angles by a mixed mechanism of microcutting and spalling [29].

The comprehensive analysis of the above research in the process of abrasive water jet processing of brittle materials is mainly to impact crack expansion and chip leading to material removal, while the processing is also accompanied by a certain amount of plastic deformation removal.

### 3.2. Processing Removal Mechanism of Plastic Materials

The mechanism of removal for plastic materials is still being refined. Ductile erosion mainly applies to soft metals or materials that can undergo significant plastic deformation. The removal mechanism for plastic materials has been generally accepted as cutting wear and deformation wear; the material removal process is generally considered to be dominated by microcutting and shaping removal of the material. When abrasive particles impact the surface at a small angle, a smooth cutting area is produced [30]; in contrast, when impacting the surface at a large angle, mainly deformation wear occurs: a surface profile with a deflected stripe shape at the bottom of the cut section [31,32], as shown in Figure 3. Thus, the material removal process can be explained by the microcutting process of the material being machined, which is similar to conventional microcutting, as shown in Figure 2b.

**Figure 5:**
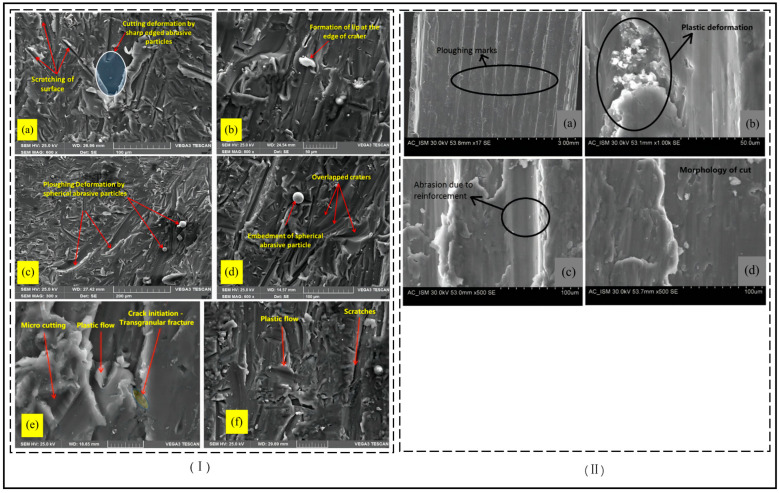
(**I**) Deformation mechanism involved in AZ91 magnesium alloy [35,36]. (**a**–**d**) indicates cutting deformation, ploughing deformation and plastic deformation of the material, (**e**,**f**) indicates micro-cutting, crack initiation and plastic flow in materials. (**II**) SEM image of the machined surface [37]. (**a**) ploughing mark,(**b**) plastic deformation; (**c**) abrasion; (**d**)morphology of cut.

In observing the cutting process of the shaped material, the researchers determined the mechanism of material removal and separation through a corresponding series of studies (usually metal materials with good ductility and plasticity) in plastic metal materials processing observed when the first shear damage occurs, so that metal chips are gradually stripped from the body of the processed material, followed by abrasive particles through further collision with the material, shear, and grinding role to complete the processed material cutting. In the process of processing, water only plays a role in the acceleration of abrasive particles and cleaning waste. Therefore, a longer process of elastic and plastic deformation is required before fracture occurs during material processing removal. Compared with the energy and time required for fractures to occur in plastic materials, the microcutting effect of abrasive particles on plastic materials will be more significant. Thus, the removal mechanism of metallic materials is considered to be based on microcutting and plastic removal [33]. Chen et al. [34] research showed that abrasive water jets exhibited major cutting mechanism removal during the machining of ductile materials such as aluminum, mild stee, and plexiglass. Niranjan et al. [35,36] reported the presence of both cutting wear and deformation wear removal mechanisms observed in cutting AZ91/Al2O3 material, and the combined removal mechanisms of ductile shear, ploughing deformation, and fracture were clearly observed in the wear trajectory of the material, as shown in Figure 5. Sasikumar et al. found the same thing when cutting aluminum-based alloy material. This is similar to the removal mechanism for other shaping materials [37]. Cao et al. [38] argue that when the jet impacts the surface of a plastic material at a vertical angle, the surface is extruded and deformed due to the compressive stress; when the surface of a plastic material is eroded at a certain angle and inclined, the abrasive particles act repeatedly on the surface of the workpiece in the form of rotary cutting or transverse ploughing, thus achieving the removal of the material being worked.

The comprehensive analysis of the above research in the process of abrasive water jet processing of plastic materials is mainly to impact generated cutting and repeated plastic deformation resulting in material removal, while the processing is also accompanied by a certain amount of microcutting to produce chip removal.

### 3.3. Processing Removal Mechanism of Composite Attribute Materials

The usual definition of material properties as brittle or plastic is somewhat limited, because some materials are neither absolutely brittle nor absolutely plastic, but under certain conditions, they undergo certain transformations with each other, making the material properties exhibit a certain complexity. At present, the research on abrasive water jet machining mechanisms is mainly aimed at the erosion model of brittle materials and plastic materials. However, in engineering practice, it is possible to encounter processed materials that satisfy the properties of both brittle and plastic materials, where the material removal process should conform to the processing and removal mechanism of a composite attribute materials. The nature of the material being processed affects the material with one of the property (brittle or plastic) removal modes being dominant and the other property removal mode being secondary, but both removal modes exist simultaneously; at the same time, the material removal mode is not entirely dependent on the material properties and can also vary with the processing parameters. Researchers in soft metal processing have found that the brittleness of the material also increases with increasing jet impact velocity, and even for hard ceramics, the toughness properties increase when processed with finer abrasive particles [39]. Wang et al. [40] developed a new universal finite element model for erosion wear with brittle and ductile material properties. The effects of impact velocity, impact angle, and particle impact force on the target are simulated; the results show that the predicted results of the established erosion model are consistent with those of the experimental analysis. Ali and Wang [41] proposed a model (Equation (1)) that applies to both brittle and plastic material removal. This model can account for the effects of impact velocity, angular variation, material properties, and particle size and shape on the machining process.

(1) $$\frac{V_{r}}{m_{p}}=\left(A_{1}\;\sin^{2}\theta +{\;A}_{3\;}\cos^{2}\theta \right)\;v^{2}+\left(A_{2\;}\sin^{3}\theta +A_{4}{\;\cos}^{3}\theta \right){\;v}^{3}$$

where

$$A_{1}=k_{1}\frac{10^{4}\;∆}{\alpha +\beta \;H},\;A_{2}=k_{2\;}\left(1-10^{4}\;∆\right){\left(\frac{d}{2\;H\;{∆}^{2}}\right)}^{3/2},\;A_{3}=A_{1}\frac{k_{3}}{k_{1}},\;A_{4}=A_{2}\frac{k_{4}}{k_{2}},\;∆=\frac{K}{\sqrt{2\;E\;H\left(1+2\;\varepsilon_{f}/\varepsilon_{e}\right)}}$$

where V_r_ is volume removed, m_p_ is the abrasive particle mass, and A_1_–A_4_ is the experimental determining factor, and its calculation is obtained using the corresponding formula. Δ is a dimensionless quantity, θ is the impact angle, v is the impact velocity of the abrasive particle, k_1_–k_4_ are experimental parameters, α and β are material constants relating dynamic to quasistatic hardness, d is the size of the abrasive particle, H is target material hardness, E is the elastic modulus of the target material, and ε_f_ and ε_e_ are fracture and elastic strains.

The removal of material in the brittle and plastic modes is usually based on the deformation of the material being processed and the extension of cracks, which subsequently leads to changes in the structure of the material being processed. However, as the abrasive particles become finer and the impact force is less than the threshold for plastic deformation of the material, only elastic interactions between the abrasive particles and the machined surface occur, and the removal mechanism changes from an indentation mechanism to a “surface area mechanism”. Peng et al. [42,43] propose an “elastic” material removal mode based on surface hydroxylation effects and chemisorption theory, which is not limited to the processing properties of the material being processed but provides a very small contact force and removes the material by interchemical collision reactions between the abrasive particles and the substrate of the material being processed. During the removal process, the atomic arrangement of the material is restored to its original state; thus, the “elastic” removal mode ensures the surface integrity of the material.

From a comprehensive analysis of the above, the abrasive water jet processing composite attribute materials removal mechanism research is scarce, and usually, composite attribute materials tend to be classified as brittle materials or plastic materials, but there is a certain difference with the practice of processing for composite attribute materials removal mechanisms, for composite attribute materials processing removal mechanism is constantly enriching and perfecting, and at the same time, attracting researchers to the study of this issue. 

## 4. Abrasive Water Jet Machining Process Simulation

With the rapid development of computer technology, numerical simulation methods have become one of the main technical means and effective methods of scientific research. With the current abrasive water jet technology in the processing process, there is no direct observation of the jet impact on the workpiece throughout the process state, for example, the energy distribution of the jet, the trajectory of the abrasive particles, the jet speed, the wear pattern of the nozzle, the evolution of the damage crushing process of the workpiece after being impacted, etc. And based on the numerical simulation method, this problem can be well solved, and at the same time, it can provide further guidance and verification for revealing the processing mechanism and the experimental process.

### 4.1. Simulation of the Fluid Motion of Abrasive Water Jet

**Figure 6:**
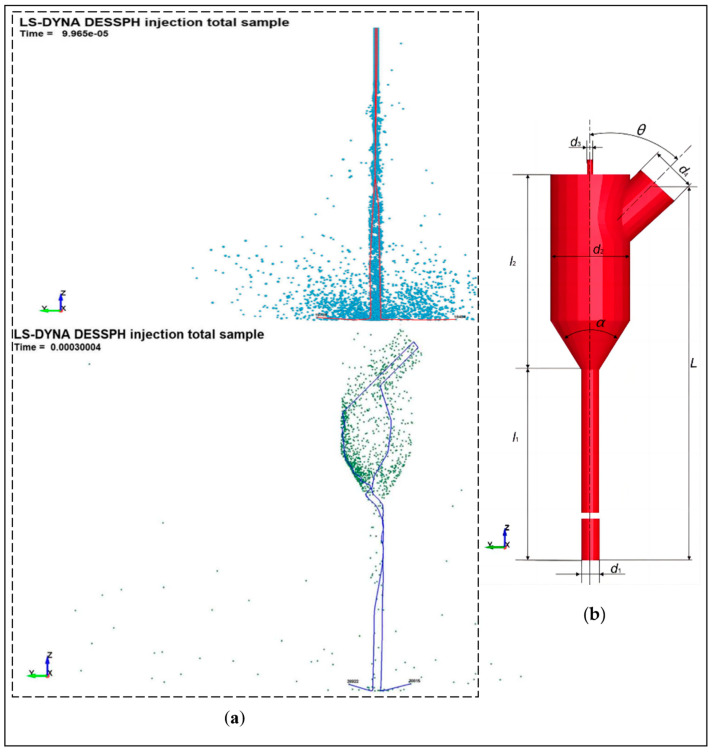
Simulation of abrasive water jet motion processes [47]: (**a**) the trajectory of the water flow and abrasive particles; (**b**) geometric modeling of the nozzle.

**Figure 7:**
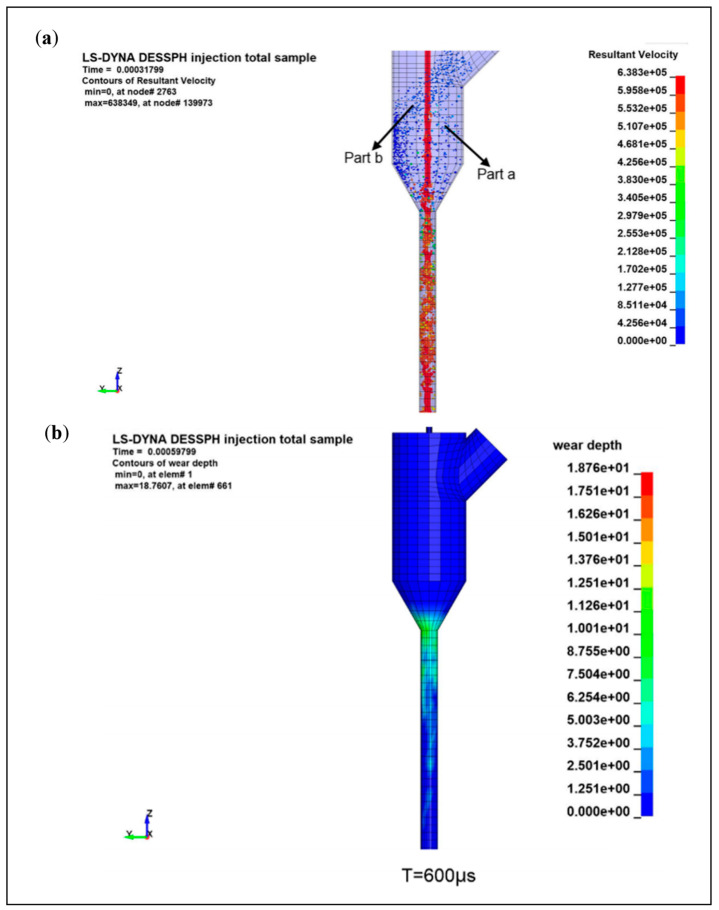
(**a**) the mixing and accelerating process of abrasive particles in the nozzle; (**b**) the wear simulation of abrasive waterjet nozzle [47].

Li et al. [44] used CFD methods to simulate the velocity field of the abrasive water jet and the trajectory of the abrasive particles and analyzed the factors affecting the service life and impact (cutting) capacity of the nozzle to obtain the optimum nozzle inlet diameter coefficient and convergence section length coefficient to provide theoretical reference for nozzle structure optimization. Deng et al. [45] did the same study and obtained the laws of jet exit velocity as influenced by the nozzle convergence angle, the length of the focusing tube, and the effect of abrasive flow rate on the wear condition of the inner wall of the nozzle. Lin et al. [46] used SPH-FEM to simulate the motion and acceleration state of abrasive particles inside the nozzle in a premixed abrasive water jet and the whole process of impacting the target body. The movement trajectory of the abrasive particles inside the nozzle and the acceleration process are revealed to be influenced law by the nozzle structure, providing a visual observation for the study of the processing of abrasive water jets, as shown in Figure 6 and Figure 7.

### 4.2. Process Simulation of Abrasive Water Jets Impacting Brittle Materials

Liu et al. [48] used the SPH method to simulate the damage and crushing process of concrete impacted by abrasive water jets and to analysis the effect of abrasive concentration on the damage and crushing efficiency of concrete under certain conditions. It was found that the concrete crushing depth and crushing efficiency were optimal when the abrasive concentration was around 20%. Zhuang et al. [49] used a coupled SPH-FEM method to simulate the cutting of steel and concrete by abrasive water jets and found that there was a difference in the cutting mechanism between the two, with the abrasive particles playing a key role in the cutting of steel, while the contribution of water is relatively limited. Compared with this, the contribution of water in the cutting of concrete is significant, as it reduces the effect of changes in traverse velocity on the depth of cut of concrete, resulting in a greater depth and width of cut in concrete than in steel. Meng et al. [50] used the ANSYS/Ls-dyna method to simulate the mechanism of rock erosion by individual abrasive particles in an abrasive water jet and simulated the effects of different sizes of abrasives, shapes, and velocities on the erosion effect of rocks, and the results of their influence factor analysis provide a reference for the optimization of abrasive water jet cutting parameters. Wei et al. [51] used coupled CFD-DEM to simulate the process of coal rock breaking by premixed abrasive water jets and investigated the influence law of abrasive mass fraction on the rock breaking effect. It was found that by increasing the mass fraction of the abrasive and controlling it within the optimum mass fraction for the specific conditions, the energy conversion efficiency and utilization rate could be improved, and the best coal rock breaking results could be obtained with lower energy consumption, and the feasibility and accuracy of the simulation was verified through rock breaking experiments. Zhou et al. [52] used the SPH method to simulate the damage evolution process of abrasive water jets impacting pore rock masses and analyzed the influence law on rock damage by adjusting the pore characteristics parameters and jet parameters. It was found that the pore size and distribution significantly affect the breakage shape of the rock mass and that the velocity of the jet and the abrasive concentration are also major factors affecting the breakage of the rock mass. Ma et al. [53] used the SPH method to simulate the three-dimensional dynamic process of rock breaking by abrasive water jet impact and simulated the effects of processing parameters on rock damage deformation and crushing, obtaining the main factors affecting the rock breaking effect. By increasing the abrasive concentration and jet velocity, the effect of rock breaking can be greatly improved. Mi et al. [54] used a coupled SPH-FEM method to simulate the postmixed abrasive water jet impact rock crushing damage process and found its influence law on rock breaking effect by adjusting parameters such as jet velocity, abrasive concentration, and rock surrounding pressure, and the rock breaking experiments were consistent with the simulation results, which verified the accuracy of the numerical simulation and provided corresponding theoretical support for the application of abrasive water jet rock breaking. Li et al. [55] used a coupled SPH-FEM method to simulate the crushing process of abrasive water jets impacting nonuniform granite and analyzed the evolution of hole depth, diameter, and erosion range during the impacting nonuniform granite process, then obtaining the error between the two within a certain range through mutual verification between experiments and numerical models, which provides a basis for studying the crushing mechanism of abrasive water jets impacting granite.

### 4.3. Process Simulation of Abrasive Water Jets Impacting Difficult-to-Process Materials

**Figure 8:**
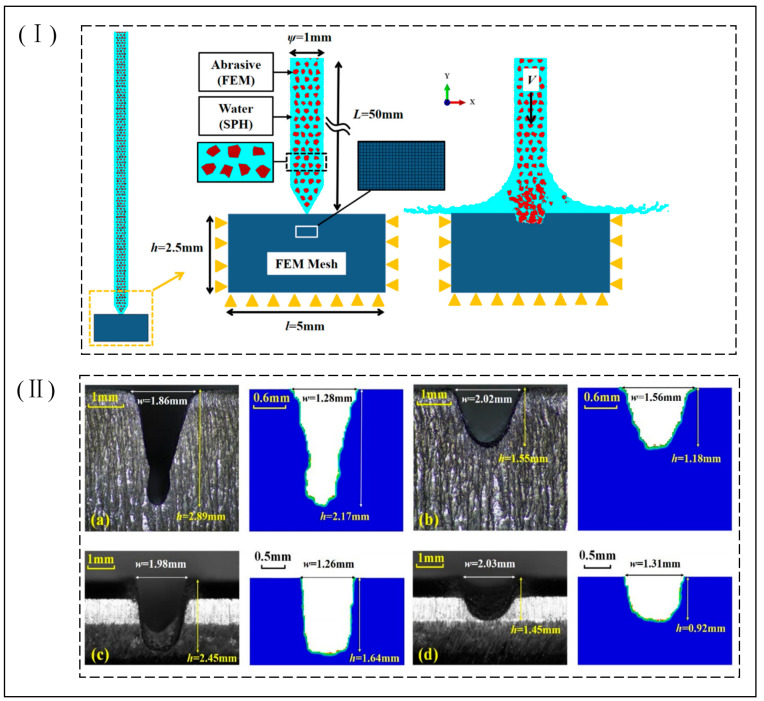
Simulation and experiment of abrasive water jet impact on workpieces [58]: (**I**) FEM-SPH coupled model of abrasive water jet machining material surface; (**II**) comparison of the experiments and simulations of the kerf profiles under different impact conditions. (**a**,**b**) are kerf profiles of Ti6Al4V, and (**c**,**d**) are kerf profiles of AISI304.

Zhigang et al. [56] used ANSYS/Ls-dyna to establish an abrasive water jet impact model for carbon composites to study of the effect of jet pressure, target distance on punching depth, and punch delamination. It was found that jet pressure is the main influencing factor for the machining depth and punching delamination, and the simulation results are consistent with the test, which verifies the feasibility and validity of the simulation model. Miao et al. [57] used the SPH-FEM coupling method and the ALE multimatter algorithm to simulate the process in the simulation of abrasive water jet erosion of stainless steel 304#; the simulation of the flow field inside the jet nozzle and the simulation of the jet erosion process were combined to achieve a full simulation of the abrasive water jet erosion process. By comparing the two simulation methods, it was found that when the abrasive content is low or high, the abrasive particle conversion method is closer to the experimental value, i.e., with the SPH-FEM method and when the abrasive content is moderate, the water conversion method is closer to the experimental value, i.e., with the ALE method; therefore, the two methods should be chosen according to the abrasive content. Du et al. [58] used the FEM-SPH method to simulate the maximum impact depth and impact edge profile of abrasive water jets impacting titanium alloys (Ti6Al4V) and stainless steels (AISI304) under different impact conditions (jet type, abrasive type, and process parameters) to analyze the influencing factors to give the key factors affecting the processing of abrasive water jets, and to experimentally and numerically simulate mutual validation, as shown in Figure 8. Du et al. [47] used the SPH-DEM-FEM method to establish a simulation of the full process of an abrasive water jet impacting workpiece C45 steel; this consists of two stages, the mixing of the high-pressure water with the abrasive particles after entering the nozzle and the impact of the jet on the workpiece after mixing, simulating the mixing and acceleration process of high-speed water and abrasive in the focus tube and the evolution of impact damage after impacting the workpiece, obtaining the key influencing factors affecting the target requirements (nozzle damage area, cutting depth, kerf characteristics, etc.) by adjusting the process parameter settings and verifying the realism of the simulation model by the corresponding experimental results.

The comprehensive analysis of the above research in the simulation of the abrasive water jet processing process is mainly focused on the use of finite element analysis (FEM) and the smooth particle hydrodynamics (SPH) coupling method to establish the model, its simulation results, and experimental results match to reveal that its abrasive water jet processing process mechanism has an important guiding significance, while the optimization of the processing target has important engineering practical significance. However, there is a certain simplification or idealization of the model building in the simulation process (e.g., the abrasive shape is treated as a spherical shape), which also affects the accuracy of the simulation effect and causes a certain error with the actual situation; therefore, in the modelling process of abrasive water jet processing, the influence of the relevant parameters and the accurate establishment of the constitutive model of the processing object should be fully considered.

## 5. Influencing Factors in the Processing Removal Process of Abrasive Water Jetting

**Figure 9:**
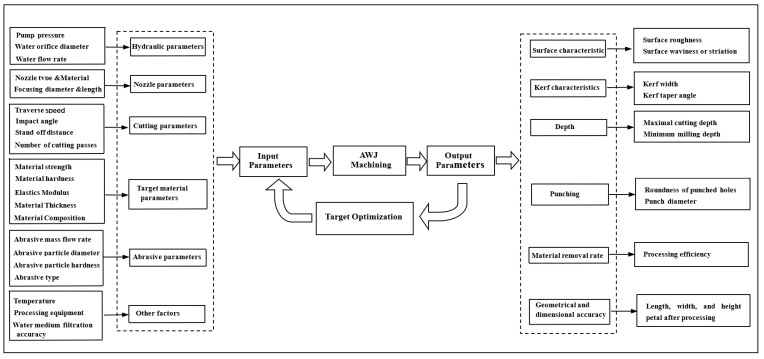
Process parameters influencing abrasive water jet machining.

The processing of abrasive water jets is usually measured by one or more target output requirements, such as depth of cut, surface quality, or processing efficiency, as the target output requirements for the object to be processed by the abrasive water jet; thus, the level of influence of the corresponding process parameters differs for different processing target requirements. The main process parameters include jet pressure, feed rate, separation distance, nozzle size, angle of incidence, abrasive type, abrasive concentration, material to be processed, and other factors, as shown in Figure 9. In the following, the maximum cutting depth and surface quality in the abrasive water jet machining process are introduced as the main processing output objectives.

### 5.1. Factors Influencing the Maximum Depth of Cut during Abrasive Water Jet Processing

**Figure 10:**
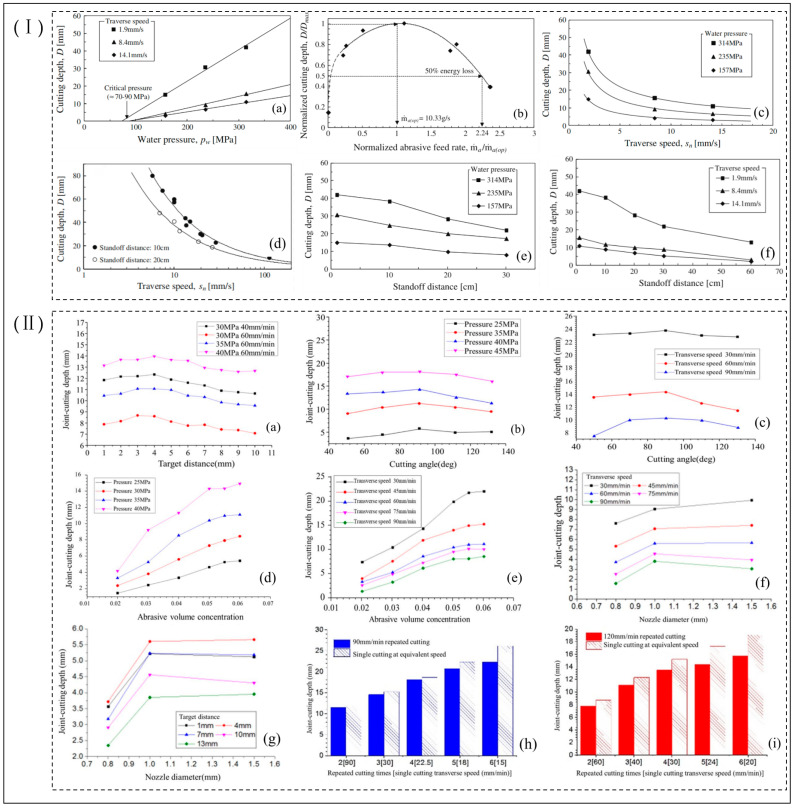
Main parameters affecting cutting depth. (**I**) (**a**) effect of the water pressure on the cutting depth; (**b**) effect of the abrasive feed rate on the cutting depth; (**c**) effect of the jet traverse speed on the cutting depth(an intensifier-type pump at standoff distance of 1 cm); (**d**) effect of the jet traverse speed on the cutting depth(a plunger-type pump at water pressure of 245 Mpa); (**e**) effect of the standoff distance on the cutting depth(different water pressures where the traverse speed is 1.9 mm/s); (**f**) effect of the standoff distance on the cutting depth(different traverse speeds where the water pressure is 314 MPa); [59]. (**II**) (**a**)Influence of jet flow field dynamics and target distance on joint-cutting depth; (**b**) Influence of cutting angle on joint-cutting depth at different pressures; (**c**) Influence of cutting angle on joint-cutting depth at different traverse speeds; (**d**) Influence of abrasive volume concentration on joint-cutting depth at different pressure; (**e**) Influence of abrasive volume concentration on joint-cutting depth at different travelling speeds; (**f**) Influence of nozzle diameter on joint-cutting depth at different travelling speeds; (**g**) Influence of nozzle diameter on joint-cutting depth at different targets distances; (**h**) Influence of nozzle diameter on joint-cutting depth at traverse speeds of 90 mm/min; (**i**) Influence of nozzle diameter on joint-cutting depth at traverse speeds of 120 mm/min. [60].

The depth of cut is usually used as a measure of cutting capacity, with the deeper the maximum depth of cut, the greater the cutting capacity and the higher the energy possessed by its jet. The influencing factor in determining the higher energy of the jet is the different combinations between the main process parameters. Researchers found a positive correlation between depth of cut and jet pressure, which increases as it increases over a range, and a negative correlation with feed rate and separation distance, which decreases as it increases; the depth of cut is also influenced by the abrasive flow rate, angle of incidence, and time of action, as shown in Figure 10 [59,60].

Researchers have conducted a great deal of research on the factors influencing the depth of cut during abrasive water jet processing. Liu et al. [47] investigated the effect of abrasive concentration on the crushing depth and damage efficiency of jet impact concrete and found that abrasive concentration was positively correlated with crushing depth and damage efficiency, increasing with increasing abrasive concentration, with a rapid increase in the range of 10–20%, reaching a maximum at around 20%. Karmiris-Obratański et al. [61] investigated the effect of the number of cuts and transverse speed on achieving greater depth of cut in abrasive water jets and found that multiple cuts resulted in better results compared with a single cut, summarizing that the transverse speed was directly proportional to the removal rate of the material being machined but inversely proportional to the depth of cut, surface roughness, and kerf taper. Arab et al. [62] studied the effect of pump pressure and transverse feed rate on the cutting effect of abrasive water jets for cutting four types of rock and found that the removal volume and cutting rate of rocks increased with the increase in pump pressure and decrease in transverse feed rate, and the best cutting efficiency was obtained when the pump pressure was 400 MPa and the transverse feed rate was 200 mm/min. The best cutting efficiency was obtained at a pump pressure of 400 MPa and a transverse feed rate of 200 mm/min. Researchers [63,64] found that jet pressure and transverse feed rate had a significant effect on material removal and depth of cut results when cutting Al-SiCp-MMCs and Ti6Al4V alloys with abrasive water jets and that large depths of cut could be achieved by selecting a combination of transverse feed rate and abrasive mass flow rate parameters within a suitable jet pressure range. Aydin et al. [65] studied the effect of jets of different types of abrasives on the performance of cutting marble; the study found that silicon carbide and fused alumina oxide abrasives showed better cutting performance in terms of depth of cut and kerf angle for the same cutting parameters. In contrast, glass bead abrasives are more likely to give a smooth cutting surface. Furthermore, there is a strong correlation between abrasive density and hardness on the cutting performance of AWJ. Karakurt et al. [66] studied abrasive water jets cutting granite and found that the abrasive particle size had an important effect on the depth of cut, finding that the depth of cut was deeper when coarser abrasive particles were used and that the depth of cut increased with increasing abrasive particle size, while the surface roughness decreases with increasing abrasive grain size. The transverse speed is the most important parameter influencing the depth of cut, while pointing out that the crystal structure and physical properties of the granite also affect its depth of cut and surface quality. Mogul et al. [67,68] established a predictive depth of cut model in the process of abrasive water jet cutting Ti6Al4V and found experimentally that the greatest influence on the depth of cut was the transverse speed, followed by the abrasive mass flow rate, feed rate, jet pressure, angle of incidence, and target distance. Jiang et al. [60] investigated the process of influence of jet pressure, transverse velocity, target distance, cutting angle, abrasive volume concentration, nozzle diameter, and number of repeated cuts on the depth of cut during cutting of Q345 steel by an abrasive water jet. The experimental study found that the depth of cut is closely related to the above-mentioned operational processing parameters, of which transverse speed, pressure, and target distance are the key parameters affecting the depth of cut, and the effect on depth of cut decreases in descending order.

The comprehensive analysis of the above shows that in the abrasive water jet process, the maximum depth of cut impact factors will vary with the materials processed, types of materials, and processing methods, and the weight and order of the influence of the process parameters on the maximum depth of cut will vary somewhat, but the correlation with the law of the influence of process parameters on depth of cut is constant.

### 5.2. Factors Influencing Surface Quality during Abrasive Water Jet Processing

The quality of the machined surface is mainly a measure of the machined effect of the object being machined, mainly through the machined surface roughness and the quality of the kerf. Researchers have found that surface roughness increases with increasing travel speed and spacing distance and decreases with increasing jet pressure. The increase in energy applied to the surface of the machined material at higher jet pressures leads to an increase in depth of cut, cutting efficiency, and material removal rate, while surface roughness and kerf taper decrease.

**Figure 11:**
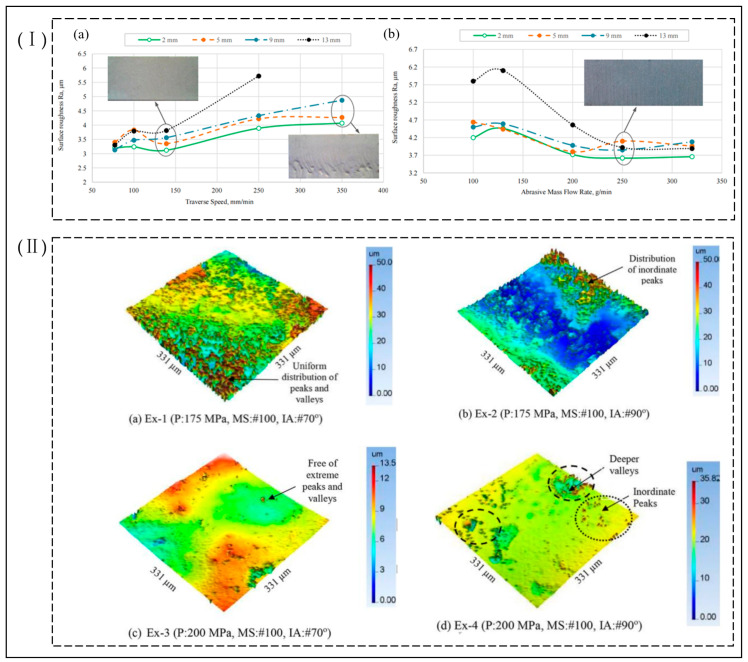
(**I**) effect of process parameters on surface roughness of different cutting areas [69], (**a**) The effect of traverse speed on the surface roughness on different zones of the cut surface—15 mm thickness; (**b**) The effect of abrasive mass flow rate on the surface roughness on different zones of the cut surface–15 mm thickness; (**II**) 3D surface topography at different cutting conditions [80].

Begic-Hajdarevic et al. [69] found that the movement speed of the jet nozzle had a large effect on the surface roughness of the lower part of the machined section, resulting in the formation of distinct machined streak marks and streak tail deflections, as shown in Figure 11Ⅰ. Sasikumar et al. [37] investigated the effect of jet pressure, travel speed, and interval distance of abrasive water jets on the kerf characteristics during processing of aluminum 7075 metal matrix composites and found that travel speed had a significant effect on the top width of the kerf, with smaller kerf characteristics obtained when the cutting effect is on the top width of the kerf, with smaller kerf characteristics obtained when cutting at a lower travel speed and smaller surface roughness, obtained when using a combination of high pressure and low travel speed. Meanwhile, researchers [70] found that variation in travel speed had a significant effect on the surface finish quality of the material and that controlling travel speed was effective in controlling surface roughness and obtaining a smaller response of the kerf features. Sharma et al. [71] found a significant effect of travel speed on surface roughness and kerf angle taper in cutting H13 die steel, as did Hascalik et al. [72] in cutting Ti6Al4V alloy. Szatkiewicz et al. [73] investigated the effect of process parameters of abrasive water jets on the cutting of stainless steel–polymer composite material. It was found that traverse speed was the most significant factor influencing roughness, followed by pressure and abrasive flow rate. Kmec et al. [74] found that smaller surface roughness could be achieved using smaller nozzle diameters, suggesting that nozzle diameter and bore diameter affect material removal rates, surface roughness, and geometric accuracy over a range of widths. Akkurt et al.’s [75,76] experiments have shown that pressure is inversely proportional to surface roughness when using different pressure jets to cut the same thickness of material, with higher pressure causing higher energy of jet impact, resulting in a smaller surface roughness and kerf taper. As the abrasive mass flow increases, the number of abrasives used to produce microcutting in the machining process increases, resulting in improved surface quality and increased machining efficiency. Researchers [77] have found that the greater the spacing between the nozzle and the workpiece being machined, the lower the velocity of the abrasive particles during jet contact with the workpiece, resulting in lower the material removal rate, surface roughness, and kerf taper. Kechagias et al. [78] studied the effectiveness of abrasive water jet process parameters with small spacing distances, low feed rates, and small diameter nozzles in reducing roughness and kerf gap widths when cutting steel plates. Yuvaraj et al. [79] found that the impact angle of the jet and the size of the abrasive grain size directly affect the cut surface roughness, kerf width, taper ratio, etc. Good roughness, small kerf taper, and streaks are obtained when cutting AA5083-H32 aluminum alloy at an impact angle of 70°. The same finding was made when cutting AISI D2 steel in terms of the quality of the three-dimensional surface profile obtained [80], as shown in Figure 11Ⅱ. Rajesh et al. [81] investigated the drilling of holes in Ti metal hybrid fiber core laminate material by abrasive water jets and found that the surface roughness was proportional to the spacing distance and travel speed and inversely proportional to the jet pressure and abrasive mass flow rate, with jet pressure having the greatest effect on surface roughness, followed by the abrasive mass flow rate. Shakouri et al. [82] investigated the use of different types of abrasive water jets (e.g., garnet, sugar, and bone powder) to cut bovine femurs; it was found that good cutting properties and surface quality were observed when cutting bovine femurs when sugar was used as an abrasive compared with other abrasives, and they pointed out that jet pressure and moving speed were the main factors affecting surface roughness and cutting quality, and that with higher pressure and lower transverse speed, better machined surface quality can be obtained.

From the comprehensive analysis of the above on the abrasive water jet process, the surface quality factors are due to the type and thickness of the processed material, processing methods, evaluation indicators, and different abrasive types; the process parameters of the degree of influence on the surface quality of the weight and order will result in some changes, but the process parameters on the surface quality of the impact of the law of correlation remain unchanged.

### 5.3. Improvement and Optimization of Abrasive Water Jet Process Parameters

Actual processing of abrasive water jets considers processing efficiency, production costs, processing quality, available equipment and processes, etc. Abrasive water jet processing involves a variety of processing parameters, and the abrasive water jet process parameters are effective factors that directly affect the processing results. Thus, the combination of process parameters can be optimized in order to significantly improve machining performance in order to obtain the most economical production processing. The optimization process is usually based on single- or multiple-target-level characteristics; for example, in abrasive water jet machining, the target-level characteristics of depth of cut and material removal rate are “the greater the better”, while the target-level characteristics of surface roughness, taper cut ratio, and kerf width are “the smaller the better”. Therefore, the combination of optimal process parameters for target-level characteristic during abrasive water jet processing will vary depending on the target requirements encountered and appropriate target decision methods need to be established.

Perec et al. [83] used fractal analysis to assess the surface quality of abrasive waterjet machined surfaces and verified the reasonableness of the fractal dimension for the evaluation of the surface quality within a certain range and provided a simple and reasonable method of evaluating the quality of cut surfaces in industrial practice, which is at the same time affected by the quality of the photographs of the surfaces used. Caydas et al. [84] obtained the best combination of cutting process parameters in the machining of aluminum alloy by using artificial neural networks (ANN) and regression analysis methods to optimize the surface roughness as the target level, that is, a combination of jet pressure, travel speed, spacing distance, abrasive particle size, and abrasive particle flow rate. Aich et al. [85] used a particle swarm technique approach to optimize the cutting depth as a single target level in the abrasive water jet cutting of borosilicate glass and obtained the best combination of cutting process parameters, that is, a combination of jet pressure, travel speed, spacing distance, and abrasive mass flow rate. Liu et al. [86] used response surface methodology to predict optimum depth of cut and surface roughness values in abrasive water jet machining of alumina ceramics; the results obtained from the corresponding experiments also match the predicted results, verifying the accuracy of the method and pointing out the significant effects of moving speed and abrasive mass flow rate on depth of cut and roughness. Azmir et al. [87] used multiple linear regression analysis in the processing of fibrous plastic composites by abrasive water jets to optimize the process parameters with surface roughness and kerf taper ratio as the target levels. It has been found that jet pressure and abrasive material type are the most significant controlling factors affecting roughness and kerf taper ratio. These two target levels can be achieved by increasing the pressure and abrasive mass flow rate and reducing the travel speed and separation distance. Santhanakumar et al. [88] used the combined gray correlation response surface method in AWJ-cut tiles to optimize the process parameters, i.e., jet pressure, transverse velocity, separation distance, abrasive size, and abrasive mass flow, at the target level of surface roughness and taper angle to obtain the optimum combination of process parameters and validate the accuracy of the method through relevant experiments. Radovanovic [89] used a multiobjective genetic algorithm (MOGA) in the processing of carbon steel S235 by abrasive water jets to optimize the process parameters with high productivity and low operating costs as the target-level characteristics and obtained the optimum combination of process parameters with a travel speed of 127 mm/min, an abrasive mass flow rate of 300 g/min, and an interval distance of 1 mm. Joel et al. [90] used the multiobjective teaching learning method in the abrasive water jet cutting of C360 brass for minimum surface roughness, maximum material removal rate, and hardness as the optimized target levels and experimentally verified that the optimum combination was an abrasive supply rate of 250 g/min, an interval distance of 2 mm, and a nozzle speed of 44 mm/min. Llanto et al. [91] used Taguchi’s S/N ratio method in the abrasive water jet cutting of austenitic stainless steel 304L for the reduction in surface roughness and the maximum material removal rate as the target-level characteristics for the cutting process parameters (jet pressure, abrasive mass flow rate, travel speed, and thickness of material being processed) were optimized to obtain the optimum combination of parameters for minimum surface roughness and maximum material removal rate, respectively. It is also pointed out that the thickness of the processed material is the most significant and influential control factor on the surface roughness and material removal rate. Radomska-Zalas et al. [92] investigated the abrasive water jet cutting of aluminum using IT-supported TOPSIS method, optimized the process parameters (transverse speed, pressure, and abrasive flow rate) with width of kerf and one of the surface roughness parameters (quality) as the optimization objective levels, and obtained the optimum combination of the process parameters, i.e., transverse speed of 0.75 mm/s, pressure of 250 MPa, abrasive flow rate of 1 g/s, width of kerf 0.75 mm, and surface roughness of 14.49 mm. Akhai et al. [93] used Taguchi’s gray relational (TGRA) analysis method in the processing of Al-6061 aluminum alloy by abrasive water jets to optimize the process parameters, with surface roughness, material removal rate, and edge width as the target-level characteristics for the process parameters (travel speed, spacing distance, and abrasive mass flow) to be optimized and obtained the optimum combination of process parameters travel speed of 100 mm/min, spacing distance of 1 mm, and abrasive mass flow of 300 g/min, indicating that the material removal rate was inversely proportional to surface roughness and edge width. Krenicky et al. [94] used a modified photographic method to optimize the cutting process parameters (abrasive mass flow, pump pressure, and travel speed) for abrasive water jet cutting of wear-resistant steel using surface roughness and abrasive water jet deflection as the target level and obtained the optimum combination of process parameters with an abrasive mass flow rate of 270 g/min, pump pressure of 380 MPa, and travel speed of 10 mm/min. Perec et al. [95] studied the optimization of process parameters (feed rate, water nozzle diameter, and concentration of abrasive) using response surface methodology with depth of cut as the target-level feature when cutting tool steel under recycled abrasive conditions and obtained the optimal combination of process parameters as follows: water nozzle diameter of 0.33 mm, feed rate of 2 mm/s, GMR80 concentration of 19.93%, and SPDG60 concentration of 20.53%, and the maximum depth of cut for GMR80 and SPDG60 abrasives was obtained as 28.39 mm and 21.98 mm, respectively. Perec [96] studied the effect of the disintegration of abrasive grains during abrasive water jet machining on the machining effect and the significance of abrasive recycling on machining; for example, the recycling of abrasives reduces material costs, and the disintegration of abrasive grains during processing produces new sharp angular surfaces, making it good for machining (reducing kerf taper and improving parallelism of the cutting surface). At the same time, recycling is beneficial to environmental protection and improving the efficiency of the cutting process, which has very important practical significance. Because the abrasive concentration has no significant effect on the degree and properties of abrasive particle disintegration within a certain range, it should be considered in the actual process optimization. Chandgude et al. [97] used an artificial neural network (ANN) coupled with the NSGA-II algorithm to optimize the process parameters (water pressure, travel speed, abrasive mass flow rate, and spacing distance) for CFRP cutting using surface roughness and corner edge width as the target-level feature. The optimum combination of process parameters was obtained as a water pressure of 313 MPa, travel speed of 196 mm/min, abrasive mass flow rate of 214 gm/min, and spacing distance of 0.5 mm. It was pointed out that the surface roughness was inversely proportional to the travel speed, abrasive mass flow rate, and spacing distance and positively proportional to the jet pressure, with the angular edge width values decreasing as the pressure and travel speed increased. Table 1 summaries in detail the methods, objects, mathematical models, and results of the optimization of target-level features in recent years.

Analysis of the above research shows (1) the processing of materials concentrated in metal materials or difficult-to-process brittle materials, such as aluminum alloys, stainless steel, ceramics or composite material. (2) Optimization objectives focus on surface quality (e.g., surface roughness, kerf taper), depth of cut, and material removal rate. (3) The process of target-level optimization concentrates on the optimal combination of key process parameters of the abrasive water jet, i.e., optimization and combination of jet pressure, abrasive mass flow rate, travel speed, and spacing distance.

The shortcomings of the above research are that (1) at present, the main focus is on the optimal combination of key parameters, ignoring the impact of the abrasive characteristic parameters themselves on the optimization, while the abrasive in the abrasive water jet in the processing process is a key factor affecting its cutting ability; at the same time, there are other processing parameters (nozzle and pressurized pipeline) which need to be considered, for example, the length and wear of the pressurized pipeline are almost ignored under the current experimental environmental conditions, but they cannot be ignored when the pipeline is long. For example, in the actual application of the project, there are cases where the length of the pipeline cannot be ignored. (2) At present, optimization is mainly performed under experimental environment conditions, ignoring the actual working environment conditions; at the same time, the application requirements under the environmental conditions of engineering practices are not comprehensively considered for the optimization objectives, for example, in the experimental environment conditions for processing quality, as the optimization goal process often ignores the processing efficiency, but in practice, production processing efficiency has an important value. At the same time, engineering practices not only consider cutting capacity but also need to consider the comprehensive energy consumption, water and material consumption, etc.; therefore, the optimization in the experimental environment cannot meet the application of engineering practices.

In future work, the process of target optimization should be combined with the needs of engineering practices and the conditions of the practical environment, because some parameters of the key process parameters have a certain fixity under the actual conditions of production. For example, the effect of standoff distance will be ignored in practice cutting because the actual machining must always be processed with the jet core area, and the change in target distance is very small, which is usually considered fixed, and this only applies to the case of small target distance. The target optimization process (e.g., multiobjective optimization) will be subject to equipment conditions, process parameters of their own adjustable range, process parameters on the processing of the influence of the law and multi-input process parameters of the conflict between each other, and so on, so that the quality of the processing may not be able to meet the requirements and thus can be selected through the use of reasonable processing strategies to assist in improving the quality of processing, such as the use of nozzle tilt cutting, multiple cutting, etc.

The comprehensive analysis of the above research on the improvement and optimization of abrasive water jet process parameters shows that it will vary with the type of material processed, processing method, and optimization target level, and the weight and order of the impact of the process parameters on the optimization target level will change accordingly, but the correlation with the impact of the process parameters on the target level law remains unchanged. Therefore, there is currently no unified standard model for the improvement and optimization of abrasive water jet processing process parameters, which adjusts the process parameters in due course as the processing requirements change, but for the same performance or type of material and the same optimized target level of processing, the optimization or improvement model established has strong guidance and generality in the corresponding range and provides a corresponding theoretical basis for the database of common models established for the classification and sub-objective optimization of processing.

## 6. Application and Development Directions of Modern Abrasive Water Jet Technology

### 6.1. Application of Abrasive Water Jets

**Figure 12:**
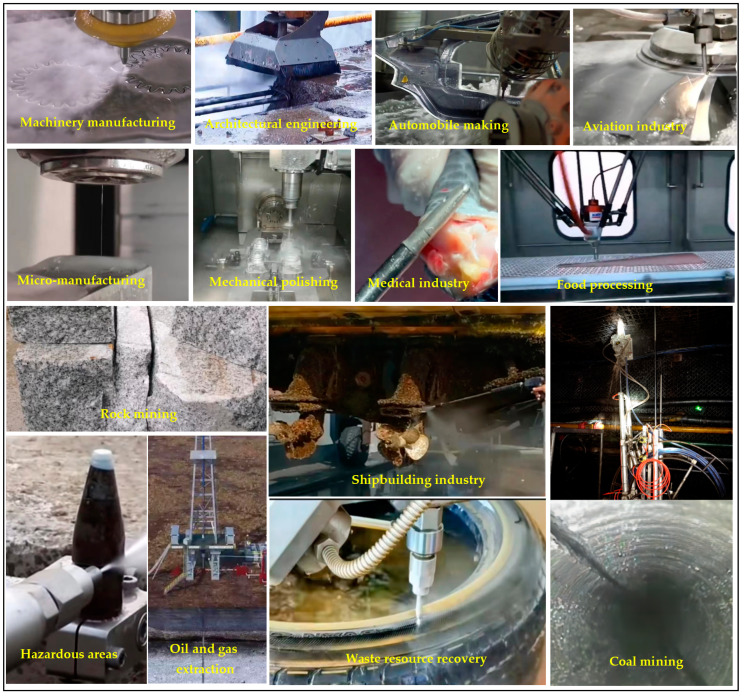
Application fields of abrasive water jets.

Abrasive water jet technology as a highly efficient, green, and environmentally friendly special processing technology [76] has strong applicability and broad prospects in the field of application, mainly including machinery manufacturing, construction engineering, automotive manufacturing, aerospace manufacturing, microelectronics manufacturing, food processing, medical industry, coal mining, oil and gas engineering, marine and ship engineering, cleaning and maintenance, and waste resource recovery [109,110,111], as shown in Figure 12. In mechanical engineering, it is more suitable for small machining points, such as plate cutting, workpiece drilling, and so on [112]. It is also capable of cutting machining with similar cutting tools, like turning, milling, and drilling [113,114]. In surface treatment, abrasive water jets are used for surface polishing, hardening, cleaning, and rust removal. In construction work and coal mining, abrasive water jets are considered to be one of the effective techniques for building crushing, rock cutting and breaking [115], and coal mining. In the medical field, jets containing substances such as salt, sugar, and bone powder as abrasives are used in orthopedic surgery, dental cleaning, etc.

Thanomputra et al. [116] proposed the harvesting of sugarcane stalks by adding salt, sugar, or ice grains as abrasives in a high-pressure water jet, with a performance that ensures more postharvest food safety and quality than traditional abrasives such as garnet. Abrasive water jets in waste recycling and hazardous material dismantling and cutting have the advantages of safety, simple process, good crushing effect, being green, and not polluting [117], for example, in ordinary scrap cutting and crushing, artillery shell cutting and dismantling, dangerous chemicals and nuclear submarines, and other dangerous occasions in the cutting and processing process where features of safety are particularly important. Abrasive water jets are often used in road and bridge repair or tunnel boring, for example, the removal of broken parts of concrete building structures by abrasive water jets, as well as crushing in conjunction with mechanical drills. In the field of coal mining, oil and gas extraction usually uses abrasive water jets in combination with mechanical equipment for rock drilling, which can effectively improve the efficiency of mining or drilling [118]. Li et al. [119] used abrasive water jets for drilling and slitting in coal mines to improve the permeability and gas collection rate of loose coal seams and achieved good results. When it comes to difficult-to-machine composites [120,121,122], abrasive water jets are well adapted to the cold processing characteristics in the cutting of composites such as carbon fiber composites, glass-fiber-reinforced plastics, and aramid fiber, which have better cut surface quality. In the processing of multidimensional surface forming materials, multiaxis machine tool abrasive water jet processing equipment can be very good for achieving processing requirements, for example, the processing of aircraft wings, fuselages, and other large-scale curved structural parts, such as rocket shells, etc. In aerospace equipment [123], aviation blades, blades, turbines, and other parts with complex curved features are increasingly used in many difficult-to-machine materials, such as carbon-fiber-reinforced epoxy resin composite materials (CFRP), metal/ceramic matrix composites (/CMC), new titanium alloys, high-temperature alloys, etc. Abrasive water jet technology is increasingly being used in the processing of these difficult materials and has become an indispensable special processing method in the field of aerospace processing. In medical applications, abrasive water jets are suitable for machining medical implant biomaterials, including bone, titanium alloys, stainless steel, and other alloys, that can achieve high surface quality in a nondestructive process without the effects of heat and pressure [124]. Abrasive water jet technology is well suited to this requirement. McGeough [125] used ice pellets as an abrasive, applying abrasive water jet technology to food processing to avoid cross-contamination between the knives used, and the ice pellets left behind at the end of the cut melted into water without affecting the quality of the food, making it safe and noncontaminating. Abrasive water jets are strongly used in the mining of minerals [126], such as coal, where the metal under the mine needs to be cut, and the abrasive water jet is able to cut without generating high temperatures and sparks, avoiding the explosion of gases caused by high temperatures and sparks.

The comprehensive analysis of the above research shows abrasive water jets’ current application is very wide, involving various industries, and that they have strong applicability, especially in heat-sensitive materials and flammable and explosive hazardous areas, where they play an irreplaceable role. Abrasive water jets in the processing process will be different depending on the industry sector or processing requirements, making the use of processing methods and abrasive types also change.

### 6.2. New Technology and Development Direction of Abrasive Water Jets

With the widespread use of abrasive water jets, researchers are paying more attention to the entire process in order to control them to achieve the desired machining target requirements. However, the development of abrasive water jet technology suffers processing mechanisms that are not clearly perceived and processing deficiencies [127,128], such as a certain phenomenon and state of affairs arising from a cutting process that cannot be clearly explained, as well as a lack of options for the rapid and optimal combination of processing process parameters for different processing target objects. Therefore, in order to improve the processing capacity, accuracy, efficiency, and range of applications of abrasive water jets and to meet the technical and application requirements of future developments in the field, the development and adoption of a number of new technologies and methods has become an effective way to address current needs. Researchers have carried out a lot of work to improve processing capacity and quality, for example, the use of forward-inclined jets for cutting in the cutting plane process, multipass cutting, and controlled nozzle oscillation are among the technical methods [129], where controlled nozzles and oscillating cutting nozzles have become one of the most effective ways to improve cutting performance without increasing process costs. Abrasive water jets are currently classified according to jet beam diameter as conventional abrasive water jets (over 500 μm), fine abrasive jets (100–300 μm), and microfabrication abrasive jets (under 100 μm) [78]. Among them, microabrasive water jet technology is the latest development of the microfabrication technology method, which is applied in the field of precision machining and micromanufacturing; it has obvious advantages in terms of processing quality, such as kerf quality, kerf width, etc., but the shortcomings are the existence of high equipment costs, processing and maintenance process with equipment processing difficulties, maintenance inconvenience, and poor stability [130,131]. Zhang et al. [132] proposed a suspended abrasive magnetic fluid jet technology based on abrasive jet technology, using electromagnetic force to effectively control the abrasive in the magnetic fluid, so that the jet has good clustering and stability, which is of great importance for the update of abrasive jet technology. Researchers have developed abrasive water suspension jet technology based on abrasive water jet technology [14,127], where the stability of the abrasive suspension is achieved by adding a quantitative amount of additives, and the abrasive suspension is pressurized by a high-pressure pump to obtain a high-velocity jet with a strong clustering to achieve the processing of the target object.

**Figure 13:**
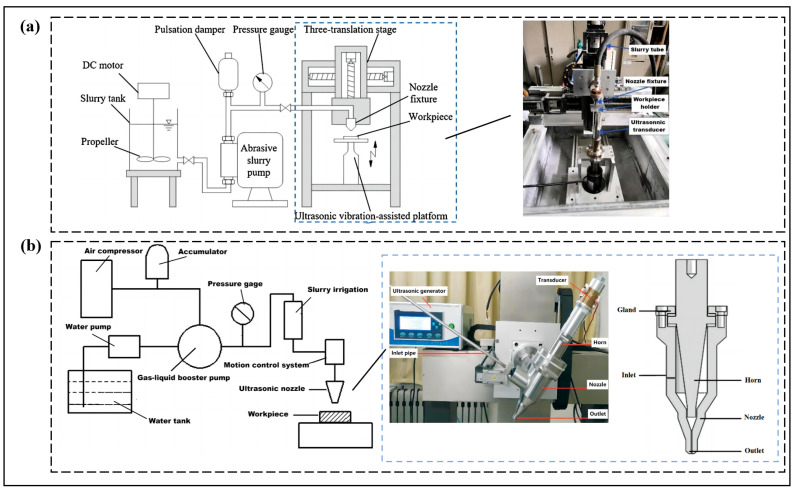
Effect of process parameters on surface roughness of different cutting areas: (**a**) ultrasonic vibrations applied to the workpiece [134]; (**b**) ultrasonic vibrations applied to the nozzle [135].

Abrasive water jet technology is gaining more and more acceptance, and its development types and application areas will become more diverse and show a trend towards integration with other technologies. This is reflected in the fact that the types of abrasive water jets will be various depending on current and future processing technology requirements, for example, there are suspended abrasive water jets, pulsed abrasive water jets, microabrasive water jets, etc. The abrasive type of abrasive water jet is diversified, and there are new types based on the requirements of the processing object and target level, which are not limited to the traditional abrasives, such as, for example, traditional abrasives (garnet, emery, iron sand, etc.) and new abrasives (ice particles, colloids, salt, bone powder, etc.). In the abrasive water jet fusion of other processing technology based on processing accuracy and processing material requirements, abrasive water jets are presented with ultrasonic processing, oscillation technology, electrolytic laser, laser processing [133,134,135], and other fusion technology for composite jet processing technology to improve its processing range, processing capacity, processing efficiency and processing accuracy, as shown in Figure 13. This is clearly becoming one of the main directions for the development of precision machining in the future. In the field of microfabrication and the processing of abrasive water jets based on processing objects and processing technology requirements, there is the development of machine tool processing equipment with micronozzles to meet the processing requirements for micromanufacturing, such as, for example, the drilling and cutting of microcomponents in integrated circuits. Abrasive water jets in these new fields show consistency with the requirements of social development and the law of science and technology development, showing high safety, high accuracy, environmental protection, and scientificity. For example, in the biological and medical field [136], abrasive water jets are used in the processing of bones or implantable biomaterials, and research into the development of safe and noncontaminating soluble abrasives (e.g., salt, sugar, and bone powder) is a new development in this direction and in the medical field. In terms of theoretical research on abrasive water jetting, researchers [127] continue to take the mechanism of material removal by AWJ processing as the main research direction to further clarify the entire processing mechanism and lay the theoretical foundation for the development of abrasive water jetting technology. For example, the integration from theory to practical application is achieved through mechanistic analysis of the removed material, numerical simulation, experimental validation, and engineering testing. Abrasive water jet technology in the future for practical use [76] presents the classification of material properties, material types, typical materials, processing methods, and optimization of target levels and requirements as guidelines for the establishment of a basic database of optimized processing parameters and processing processes according to the processing materials and requirements of intelligent control to select the database parameters with which to adapt, as well as feedback through the quality of online monitoring systems to meet their processing needs.

## 7. Concluding Remarks

Abrasive water jets are a high-efficiency, environmentally friendly processing technology, with such nontraditional processing technology has a very significant advantage for becoming one of the main technical means of future processing. This paper firstly provides an overview of the basic principles and characteristics of abrasive water jet processing technology in order to better understand and apply them; secondly, the erosion mechanism of abrasive water jet machining materials is described, and the simulation of the machining process is analyzed to have an intuitive and in-depth understanding of the machining process; then, it analyses the influence of process parameters on the output target requirements in abrasive water jet processing and removal, with emphasis on the factors affecting the depth of cut and removal rate of the processed surface. The impact of process parameters on the output target requirements in abrasive water jet machining is then analyzed, with a focus on the factors affecting the depth of cut and the removal rate of the machined surface quality, as well as the improvement and optimization of process parameters. Finally, the current status of the application of abrasive water jet technology and the new technologies and future development directions are introduced.

According to the authors’ knowledge, the following aspects of abrasive jet processing technology need to be studied urgently:

- The abrasive water jet processing mechanism should focus on the removal mechanism of composite attribute materials for in-depth research to adapt to the composite attribute materials and new materials processing removal needs.
- In the simulation of the abrasive water jet machining process, attention should be paid to the establishment of an accurate constitutive model, so that the real machining process is correctly reflected in the computer simulation; therefore, the accuracy of model establishment should fully consider the influence of relevant parameters.
- The improvement and optimization of abrasive water jet process parameters should focus on combining production practice environmental conditions on the target-level optimization to adapt to production needs, because the weight or order of the influence of the process parameters on the optimization target level may vary accordingly depending on the processing of different materials, but the correlation with the law of the influence of the process parameters on the target level remains unchanged; therefore, the establishment of the same performance type of materials and the same target level of optimization or improvement model in the corresponding range (especially in the actual production) has strong guidance and versatility.
- The application of modern abrasive water jet technology and the direction of development should focus on precision machining or microfabrication-scale direction change to adapt to growing precision in processing and refinement in demand; at the same time, it should pay attention to the integration with other technologies to form a composite processing technology to meet the improvement of its processing range, processing capacity, machining efficiency, and machining accuracy, which has become one of the main directions for the development of precision machining or composite machining in the future.

## Figures and Tables

**Table 1:** A list of several studies using various optimization techniques in the abrasive water jet machining process (2020–2022).

Optimization Type	Author	Control Parameters	Optimization Objective	Materials	Method	Optimization Results
Depth of cut	Perec A [98]	Pressure (*p*) Transverse speed (S_r_) Abrasive flow rate (m_a_)	D SR	Hardox Steel	Response surface method	The optimum control parameters: *p* = 385 MPa, Sr = 100 mm/min, m_a_ = 416 g/min, maximum depth of cut and low surface roughness of 20.66 mm and 4.23 µm, respectively. The accuracy of the modeling was verified through experiments.
	Perec A [99]	Garnet rate (G) Pressure (*p*) Feed rate (F)	D SR KA	Hardox steel	Entropy-VIKOR Approach	The optimum control parameters: G = 350 g/min, *p* = 400 MPa, F = 100 mm/min, cutting depth of 21.06 mm, roughness of 4.21 µm, kerf angle of 2.8°. The accuracy of the modeling was verified through experiments.
	Wan L [68]	Jet pressure (*p*) Mass flow rate (ṁ) Standoff distance (D) Jet angle (α) Traverse speed (u) Feed rate (S)	D MRR SR	Ti6Al4V	ADM-MO-Jaya	The optimum control parameters: *p* = 223.774 MPa, ṁ = 760 g/min, D = 21.629, α = 30.065°, u = 30.454 mm/min and S = 0.402 mm. The accuracy of the modeling was verified through experiment.
Surface quality	Yang X [100]	Cutting speed (A) Hydraulic pressure (B) Standoff distance (C)	Ta SR	CFRP	Response surface method	The optimum control parameters: A = 360 mm/min, B = 400 MPa, C = 2 mm, minimum taper error of 0.058 mm; A = 120 mm/min, B = 400 MPa, C = 2 mm, superior surface roughness of 3.58 μm. The accuracy of the modeling was verified through experiments.
	Venkateshw-ar Reddy P [101]	Standoff distance (A) Transverse speed (B) Sand flow rate (C)	MRR SR KW	Inconel-625	WASPAS and MOORA	The optimum control parameters: A = 1 mm, B = 146 mm/min. C = 250 g/min, maximum material removal rate, low surface roughness, minimum and kerf widths of 13.56 mm^3^/min, 5.10 µm, and 0.72 mm, respectively. The accuracy of the modeling was verified through experiments.
	Kant R [102]	Pressure (*p*) Abrasive mass flow rate (m) Traverse speed (TS) Standoff distance (SOD)	T SR H	EN31	Taguchi approach and Analysis of Variance	The optimal optimization results: machining time of 36 s, surface roughness of 1.59 µm, and hardness of 41.7 HRC. The accuracy of the modeling was verified through experiments.
	Karthik K [103]	Water jet pressure (A) Feed rate (B) Abrasive flow rate (C)	MRR KW	Steel 304	Grey Relational Analysis and Response	The optimum control parameters: A = 121.76 MPa, B = 80 mm/min, C = 350 g/min, maximum material removal rate and kerf width of 931.19 mm^3^/min and 1.2044 mm, respectively. The accuracy of the modeling was verified through experiments.
	Fuse K [104]	Traverse speed (T_V_) Abrasive mass flow rate (A_f_) Standoff distance (S_d_)	MRR SR KA	Ti6Al4V	Heat transfer search algorithm and RSM	The optimum control parameters: T_V_ = 193 mm/min, A_f_ = 500 g/min, S_d_ = 1.98 mm., maximum material removal rate, low surface roughness, and minimum kerf taper angles of 0.2133 g/min, 3.50 µm, and 1.98°, respectively. The accuracy of the modeling was verified through experiments.
	Rajesh M [105]	Water pressure (*p*) Nozzle distance (N_d_) Feed rate (F_R_) Abrasive flow rate (A_FR_)	SR	Flax fiber	Analysis of variance	The optimum control parameters: *p* = 310 MPa, F_R_ = 125 mm/min, N_d_ = 2 mm, and A_FR_ = 225 g/min. Minimum surface roughness of 3.04 µm obtained. The accuracy of the modeling was verified through experiments.
	Rana M [106]	Standoff distance (SOD) Abrasive mass flow rate (AMF) Transverse speed (TS)	MRR SR KA	Inconel 625	TGRA	The optimum control parameters: pressure of 310 MPa, transverse speed of 100 mm/min, SOD = 1 mm, and AMF = 300 g/min. Maximum material removal rate, low surface roughness, and minimum kerf of 25.2 g/min, 2.31 µm, and 0.79°, respectively. The accuracy of the modeling was verified through experiments.
	Wang Z [107]	Jet pressure (X_1_) Abrasive concentration (X_2_) Sprinkler angle (X_3_)	MRR	W7 diamond FAP	Response surface method	The optimum control parameters: X_1_ = 3.8 MPa, X_2_ = 3%, and X_3_ = 73°. The optimal removal rate obtained was 464.57 nm/min. The accuracy of the modeling was verified through experiments.
	Srirangarajalu N [108]	Traverse speed (T_SP_) Abrasive mass flow rate (AA_FR_) Abrasive aqua jet pressure (AA_JP_) Gap distance (G_d_)	SR KA MRR	Inconel-625	RSM-CCD	The optimum control parameters: T_SP_ = 75 mm/min, AA_FR_ = 0.55 kg/min, AA_JP_ = 300 MPa, G_d_ = 2.4 mm. Maximum material removal rate, low surface roughness, and minimum kerf angle of 141.78 g/min, 3.15 µm, and 1.44°, respectively. The accuracy of the modeling was verified through experiments.

Note: surface roughness (SR), material removal rate (MRR), cutting depth (D), machining time (T), hardness (H), taper (Ta), kerf angle (KA), kerf width (KW).

## Data Availability

Not applicable.
